# Cryo-EM Structure of a Novel Calicivirus, Tulane Virus

**DOI:** 10.1371/journal.pone.0059817

**Published:** 2013-03-22

**Authors:** Guimei Yu, Dongsheng Zhang, Fei Guo, Ming Tan, Xi Jiang, Wen Jiang

**Affiliations:** 1 Markey Center for Structural Biology, Department of Biological Science, Purdue University, West Lafayette, Indiana, United States of America; 2 Divisions of Infectious Diseases, Cincinnati Children's Hospital Medical Center, Cincinnati, Ohio, United States of America; 3 Department of Pediatrics, University of Cincinnati College of Medicine, Cincinnati, Ohio, United States of America; Tulane University, United States of America

## Abstract

Tulane virus (TV) is a newly isolated cultivatable calicivirus that infects juvenile rhesus macaques. Here we report a 6.3 Å resolution cryo-electron microscopy structure of the TV virion. The TV virion is about 400 Å in diameter and consists of a T = 3 icosahedral protein capsid enclosing the RNA genome. 180 copies of the major capsid protein VP1 (∼57 KDa) are organized into two types of dimers A/B and C/C and form a thin, smooth shell studded with 90 dimeric protrusions. The overall capsid organization and the capsid protein fold of TV closely resemble that of other caliciviruses, especially of human Norwalk virus, the prototype human norovirus. These close structural similarities support TV as an attractive surrogate for the non-cultivatable human noroviruses. The most distinctive feature of TV is that its C/C dimers are in a highly flexible conformation with significantly reduced interactions between the shell (S) domain and the protruding (P) domain of VP1. A comparative structural analysis indicated that the P domains of TV C/C dimers were much more flexible than those of other caliciviruses. These observations, combined with previous studies on other caliciviruses, led us to hypothesize that the enhanced flexibility of C/C dimer P domains are likely required for efficient calicivirus-host cell interactions and the consequent uncoating and genome release. Residues in the S-P1 hinge between the S and P domain may play a critical role in the flexibility of P domains of C/C dimers.

## Introduction

The virus family *Caliciviridae* consists of four major genera, *Norovirus*, *Sapovirus*, *Vesivirus* and *Lagovirus*. Tulane virus (TV) infecting rhesus monkeys was isolated in 2008 and proposed as a new genus, *Recovirus*, of the *Caliciviridae* family [1]. The common symptoms observed during calicivirus infection include respiratory infections, vesicular lesions, gastroenteritis and hemorrhagic disease. The noroviruses (NoVs) and sapoviruses, often referred to as human caliciviruses, are the main cause of acute gastroenteritis outbreaks in humans. Our understanding of the life cycle of human caliciviruses, including cellular entry, uncoating and assembly, has been hampered by the lack of robust tissue culture systems.

Since the first primate calicivirus structure was reported in 1994 [2], several virus-like particle (VLP) and virion structures of caliciviruses have been determined. These include the X-ray crystallographic structures of recombinant Norwalk Virus (rNV) [3] from the *Norovirus* genus, a native San Miguel sea lion virus (SMSV) [4] and a feline calicivirus virion (FCV) [5] from *Vesivirus* genus, and cryo-electron microscopy (cryo-EM) structures of recombinant Grimsby virus [6], recombinant Parkville virus [6], Rabbit hemorrhagic disease virus (RHDV, both VLPs and virions) [7], [8], [9], [10], murine norovirus (MNV) virion [9], [11] and a genogroup II genotype 10 (GII.10) NoV VLP [12]. All these structures exhibit characteristic similar overall organization: 180 capsid proteins are organized into 90 dimers, which form a T = 3 icosahedral capsid with 32 hollows around the icosahedral 3- and 5-fold axes. These structures also reveal that the capsid protein of calicivirus is organized into two major domains, the shell (S) domain and the protruding (P) domain. The S domains, with an eight-stranded jellyroll fold, are involved in the formation of the icosahedral shell, while the P domains, which can be further divided into two subdomains, P1 and P2, emanate from the shell domain and form the dimeric protrusions (P dimers) on the viral surface. This unique domain organization results in the characteristic “arch-like” densities observed on calicivirus surface. The surface P dimers are suggested to be responsible for viral-host interaction [13], [14].

TV is a small non-enveloped icosahedral virus with a positive-sense, single-stranded RNA genome of ∼6.7 kb. The complete genome of TV is organized into three open reading frames (ORFs), resembling that of *Norovirus* and *Vesivirus* but different from *Sapovirus* and *Lagovirus* which have two major ORFs. TV ORF1 encodes six nonstructural proteins, while the major structural protein (the capsid protein VP1) and the putative minor structural protein (VP2) are encoded by ORF2 and ORF3, respectively. Phylogenetic analysis has indicated that TV, the prototype virus of the new *Recovirus* genus of *Caliciviridae*, is genetically more closely related to NoVs than to other caliciviruses [1]. TV recognizes human histo-blood group antigens (HBGAs) [15], which are also receptors for human NoVs [16], [17]. The success in propagation of TV in the LLC-MK2 cell line [1], [18] suggests that TV might be a promising model system for the studies of non-cultivatable human NoVs.

Although the genome organization and protein content of TV are known, few details are understood regarding its structural properties. Here we report the cryo-EM structure of TV at 6.3 Å resolution. Structure comparisons between TV and other calicivirus members reveal that TV is structurally most similar to NoVs. Unlike most of the other known caliciviruses, an extremely flexible conformation was observed in the P domains of TV C/C dimers. These conserved and distinct structural features invite further studies of TV and its feasibility as a surrogate for human NoVs.

## Materials and Methods

### Sample Preparation

TV was propagated in LLC-MK2 cells and purified using the cesium chloride (CsCl) gradient method described previously [1] with some modifications. Briefly, LLC-MK2 cells (ATCC, Manassas, VA) were cultured with media 199 (Mediatech, Manassas, VA) to 80–90% confluence in roller bottles. The cells were then inoculated with TV at a multiplicity of infection (MOI) of 0.4. The TV-infected LLC-MK2 cell cultures were harvested after 72 hours of incubation, followed by three freeze-thaw cycles to release the viruses. The total cell culture samples were then centrifuged at 10,000 g for 10 min to remove cell debris. The viruses in the supernatants were further concentrated 40 times in dialysis tubes against PEG-8000 before being centrifuged in a CsCl density gradient at 288,000×g for 40 h (SW41Ti rotor, Beckman, Danvers, MA). The fractions containing TV were identified and quantified by RT-PCR (data not shown), SDS-PAGE, Western-blot, virus plaque assay and haemagglutination assay (HA) [19] (Figure S1).

### Cryo-EM and 3D Reconstruction

Purified TV virion samples were plunge-frozen and imaged using a Titan Krios cryo-electron microscope with field emission gun operated at 300 kV, liquid N_2_ temperature. Images were collected at 37 K nominal magnification with a dose of 25 e/Å^2^ on Kodak SO163 photographic films. The films were then digitized using a Nikon SuperCoolScan9000 scanner with a final calibrated sampling of 1.74 Å/pixel. 4702 particles were selected using combined automated selection using the *ethan* program [20] and manual screening with the *boxer* program in EMAN [21]. The microscope contrast transfer function (CTF) parameters for each micrograph were first determined using an automated fitting method [22] and then manually verified/corrected using EMAN *ctfit* graphic program. The entire TV dataset was then divided into two half datasets and processed independently for all the subsequent steps including construction of initial model, 2-D alignment and 3-D reconstruction. *De novo* initial models were constructed using the “random model” method in which random particle orientations were assigned and subsequently refined iteratively until convergence. The orientation and center of particles were determined using “projection matching” method and 3-D models were built with the direct Fourier inversion method. CTF phase correction was performed in both 2-D alignment and 3-D reconstruction and CTF amplitude weighted correction was done during 3-D reconstruction. The initial model building and iterative refinement process including particle 2-D alignment and 3-D icosahedral reconstruction were performed using an in-house developed program *jspr.py* utilizing the EMAN/EMAN2 programs and library functions [21], [23]. The Fourier shell correlation (FSC) between TV structures built from the two independently processed half datasets was then calculated to estimate the resolution. 6.3 Å, 6.9 Å and 7.9 Å were determined based on 0.143 cutoff [24], the 0.3 cutoff and the 0.5 cutoff criteria [25], respectively. Unlike almost all the previously reported single particle cryo-EM reconstructions in which the entire datasets were split after the final iteration of 2-D alignment, we split the dataset before initial model building and the iterative 2-D alignments. These two half datasets were never mixed after the split and their reconstructions were completely independent. Therefore, this reconstruction strategy is referred to as “truly independent reconstruction”. This strategy was adopted to cross-validate the reconstructions and to avoid overfitting and overestimation of resolution often associated with iterative refinements using common model for entire dataset [26], [27]. With a FSC curve calculated from two truly independent reconstructions as in our image processing strategy, the 0.143 cutoff criterion would properly estimate the true resolution of our TV map [24], [27]. After removing the particles with unstable refinement parameters, the final TV density map was constructed from the full dataset with 4338 particles by pooling the two half datasets. The final density map was sharpened first using an inverse Gaussian filter to boost the high-resolution Fourier amplitudes with reference to the computed structure factor of TV. A low pass filter derived from the FSC curve was then used to suppress the high-resolution noise and dampen the high-resolution Fourier amplitudes to the level appropriate for the resolution of the map using Henderson’s approach [24]. The map has been deposited to the Electron Microscopy Data Bank with an accession code of 5529.

### Fitting of rNV and SMSV Crystal Structures into TV Density

Crystal structures of rNV (PDB ID: 1ihm) and SMSV (PDB ID: 2gh8) were fitted into the TV cryo-EM density using UCSF Chimera [28]. All these structures were first fitted into TV with the asymmetric unit as a whole rigid body and then the fitting was refined for individual chains (A, B and C). Individual chains were further split into S domain, P1 and P2 subdomains to locally refine the fitting. The “sequential fitting” command was then applied to reduce clashes between the P1 and P2 subdomains. The S domain, P1 and P2 subdomains from both rNV and SMSV agreed well with the TV A and B subunits density (correlation coefficients of rNV S, P1 and P2 with TV: 0.73/0.79 (before/after removing the NTA of rNV S domain), 0.78, 0.81; correlation coefficients of SMSV S, P1 and P2 with TV: 0.69/0.75 (before/after removing the NTA of SMSV S domain), 0.74, 0.66). The S domain of TV C subunit also matched well with that of rNV and SMSV, but no reliable fitting was achieved between the P domain of TV C subunit and any known calicivirus crystal structures. Map segmentation was performed using the “Subregion selection” and “Color Zone” functions of UCSF Chimera.

### Fitting of the S Domains in rNV A, B and C Subunits into the S Domain Layer of TV cryo-EM Density

The S domains of rNV A, B and C subunits (PDB ID: 1ihm) within an asymmetric unit were first fitted into the corresponding density in TV in Chimera [28]. The asymmetric unit was then split into individual chains (A, B and C) and fitted into TV independently. Symmetry-related mates of A, B and C were generated using the “sym” command in Chimera, and the fitting was further refined with the constraint of icosahedral symmetry using the “symmetric fitting” function in Chimera.

### Sequence Alignment

Sequences of TV (GenBank sequence: ACB38132.1) and rNV (PDB ID: 1ihm) were aligned using the PROMALS3D multiple sequence alignment server, which uses predicted secondary structures and known 3-D structures as constraints [29]. ClustalW2 online server [30] was used for multiple sequence alignment of the S-P1 hinge between the S and P1 domains of the following caliciviruses: GenBank sequences ACB38132.1 (TV), AAC61759.1 (Pan-1 primate calicivirus), BAL60900.1 (MNV, GV.1), AAA59229.1 (Norwalk Virus, GI.1), ABV21819.2 (GII.4), ACC69026.1 (GII.11), AFN61315.1 (GIV.1), ABR15783.1 (GIV.2), AAA16220.1 (SMSV), AAA74416.1 (FCV), AAB60714.1 (RHDV).

### Generation of rNV Density Map and Low-pass Filtering of Maps

The 6.3 Å map of TV was filtered to 10 Å using a Gaussian low-pass filter in the EMAN2 *e2proc3d.py* program [23]. The 10 Å map of rNV was generated from the X-ray structure coordinates (PDB ID: 1ihm) using the EMAN *pdb2mrc* program. Maps of GII.10 NoV (EMDB: 5374), RHDV (EMDB: 5131) and FCV (EMDB: 1942) were downloaded from EM Databank (map for MNV-1 [9] is not available in EMDB). In order to make a fair comparison, the 1-D structure factor of RHDV (EMDB: 5131, with a claimed resolution of 10.5 Å) was calculated using the “calcsf” command in EMAN2 *e2proc3d.py* program, and was then applied to other maps using the “setsf” command in *e2proc3d.py* to set these maps at the same filtering level.

## Results

### TV Shares Similar Overall Architecture with Other Caliciviruses

**Figure 1:**
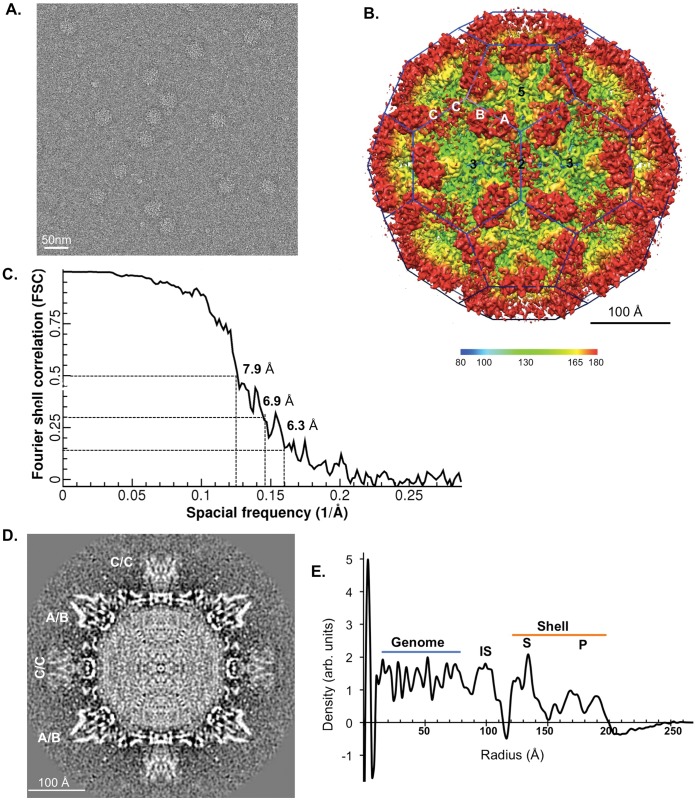
Cryo-electron microscopy and 3-D reconstruction of the TV virion. (**A**) Cryo-EM micrograph of purified TV virion sample. Scale bar: 50 nm. (**B**) Surface representation of the reconstructed TV map with the icosahedral 5-, 3- and 2-fold axes labeled. Two kinds of capsid dimers A/B and C/C are arranged on a T = 3 icosahedral cage (colored in blue). The map was colored radially according to the color key. The calculated Fourier shell correlation (FSC) is displayed in (**C**). Three resolution estimation standards in the cryo-EM field-the 0.5 and 0.3 cutoff criteria giving an estimation of 7.9 Å and 6.9 Å, respectively, and the 0.143 cutoff criterion showing an estimation of 6.3 Å- are presented. The central section perpendicular to the 2-fold axis of TV map is displayed in (**D**). (**E**) The radial density profile calculated from the 3D density map of the TV virion. Multiple density layers in TV virion structure are denoted as: “P” for protruding domain, “S” for icosahedral shell domain, and “IS” for an inner shell between the icosahedral shell and the RNA genome.

The TV structure was reconstructed from 4338 particles embedded in vitreous ice (Figure 1A and 1B). The resolution of the 3D reconstruction was estimated to be 6.3 Å based on the Fourier shell correlation (FSC) 0.143 cutoff criterion for truly independent reconstructions (Figure 1C, see more details in Methods). TV consisted of 90 dimers of the major capsid proteins (VP1), which were arranged on a T = 3 icosahedral lattice (Figure 1B). These 90 dimers were divided into two classes, denoted as the A/B dimer and the C/C dimer following the conventional nomenclature for caliciviruses. The 60 A/B dimers were located around the icosahedral five-fold axes and the 30 C/C dimers were positioned on the icosahedral two-fold axes. Such an organization of the A/B and C/C dimers resulted in a smooth shell formed by the S domains, studded with arch-like P domain protrusions of A/B and C/C dimers and the formation of 32 large hollows around the 5- and 3-fold axes. Both the large hollows and the arch-like protrusions are the most characteristic features of the calicivirus architecture. Interestingly, we observed that densities for the P domains of C/C dimers were significantly weaker and more smeared compared to the A/B dimers (Figure 1B and 1D), which indicated that the P domains of C/C dimers in TV were likely to be more flexible than the A/B dimers. Under the icosahedral shell formed by the major capsid proteins VP1 in the radii range of 118 Å to 200Å, two more density layers could be observed from the calculated radial density profile of the TV map (Figure 1E). The layer in the radii of 90–114 Å is called inner shell (IS), and has also been observed in other calicivirus virions [2], [6], [8] but not in VLPs. The composition of the IS layer has not yet been clearly elucidated. The innermost densities, located in the radii from the center to 87 Å, are presumably from the RNA genome. The capsid protein layer, the IS layer, and the genome form the whole TV virion with an approximate diameter of 400 Å. With these structural features described above, TV clearly shares the characteristic architecture of other caliciviruses. Additionally, a minor protein at ∼25 kDa was detected by the SDS-PAGE (Figure S1), which could be the minor structural protein VP2 of TV. However, the minor structural protein could be resolved in neither TV structure nor structures of other caliciviruses with confirmed existence of VP2 [31], [32], [33], probably due to the small amount of VP2 proteins and the lack of 60-fold symmetry in their arrangement within the calicivirus particles. Current knowledge on the structure and function of VP2 is limited and more studies are required to fully elucidate its roles in calicivirus life cycle.

### The Fold of the Major Capsid Protein (VP1) of TV

**Figure 2:**
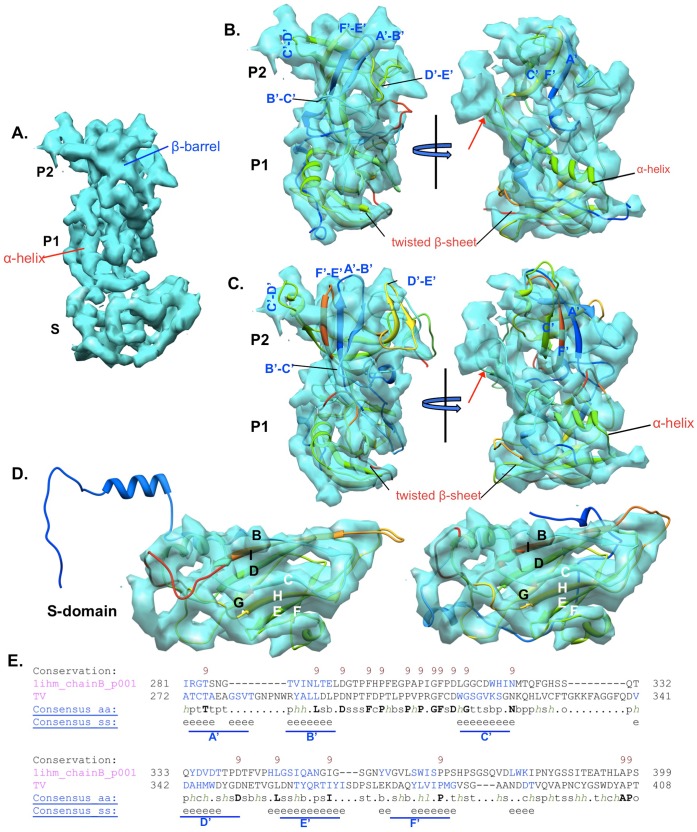
Domain organization and fold of TV capsid protein VP1. (**A**) Density of TV subunit B. S, P1 and P2 indicate the S domain, P1 and P2 subdomains. The density belonging to one conserved α-helix in the P1 subdomain and the density corresponding to the β-barrel structure at the P2 subdomain, are labeled. (**B**) The independent fitting of rNV P1 and P2 subdomains into TV, displayed in two different views. (**C**) The independent fitting of SMSV P1 and P2 subdomains into TV, at two same views as in (B). In both (B) and (C), the conserved α-helix and the twisted four-stranded β-sheet in P1 subdomain are labeled. The red arrows point to the P1-interface loop. The loops (A’–B’, B’–C’, C’–D’, D’–E’ and E’–F’) connecting the six conserved β-strands in P2 subdomain are indicated. (**D**) The S domains of rNV and SMSV were fitted into the TV density, respectively. Fitting with SMSV is displayed in the left of (D), and the right panel shows the fitting with rNV. The eight conserved β-strands in the S domain are denoted as B-I. Strands B, I, D and G form one β sheet and C, H, E, F compose the other β sheet. (**E**) Secondary structure based sequence alignment of TV and rNV. Only the P2 subdomain sequences (TV: 272–408, NV: 281–399) are shown. Positions with a conservation index above 8 are labeled in the first line of each block. The consensus amino acids are indicated with symbols: bold and uppercase letter for conserved residues; “b” for bulky residues; “c” for charged residues; “h”, “l” and “p” for hydrophobic, aliphatic and polar residues, respectively; “o” for residues with hydroxyl; “s” and “t” for small and tiny residues. The predicted β-strands (labeled with “e”) agreed well with the six β-strands (A’–F’) seen in rNV crystal structure.

Density for the major capsid protein monomer (VP1) of TV was segmented from the map and fitted with two representative x-ray crystallography structures of caliciviruses: rNV (PDB ID: 1ihmB) from *Norovirus* genus and SMSV (PDB ID: 2gh8B) from *Vesivirus* genus. As shown in Figure 2, TV shares the conserved modular S-P1-P2 domain organization of calicivirus capsid protein. In the TV S domain region, the two parallel sheet-like densities fit well with the two highly conserved BIDG and CHEF β sheets formed by eight β strands (denoted as B-I) of the jellyroll fold in the S domain of both rNV and SMSV (Figure 2D). This suggests that TV adopts the same jellyroll fold in its S domain as with other caliciviruses [3], [4], [5] and many other small RNA viruses [34], [35]. As revealed by the atomic calicivirus structures [3], [4], [5], the P1 subdomain contains two noncontiguous segments, between which the polypeptide for P2 subdomain is inserted. In the C-terminal segment of P1 subdomain there are two β strands connected by a loop (the P1-interface loop) extending towards the external surface, followed by a α helix and then a twisted four-stranded β sheet, all of which comprise the typical fold of the calicivirus P1 subdomain. Despite minor divergence in the arrangement of the aforementioned β strands and the α helix (Figure 2B, 2C), the TV P1 subdomain, in general, followed the characteristic fold of other caliciviruses at the P1 subdomain. Considering the high structural similarity exhibited in both the S domain and the P1 subdomain, and also the barrel-like density observed in the P2 segment of TV (Figure 2A), TV most likely had the P2 subdomain folded into a β-barrel composed of six β strands (denoted as A’–F’) similar to other caliciviruses. On the other hand, differences between TV and other caliciviruses (rNV and SMSV) were also observed with the greatest structural divergence seen in the P2 subdomain. This was consistent with the sequence comparison between the capsid proteins of distinct calicivirus strains, considering the P2 subdomain is a highly variable region that interacts with diverse hosts [36], [37]. After fitting of the rNV P2 subdomain, TV still had some small unoccupied densities on the external surface near the A’–B’ and E’–F’ loop region and also at the dimeric interface close to the C’–D’ loop (Figure 2B). Secondary structure based sequence alignment of TV and rNV indicated longer A’–B’ and C’–D’ loops in TV (Figure 2E), which could be responsible for the densities in the TV P2 subdomain unoccupied by the fitted rNV. The fitting of SMSV P2 subdomain into TV density is noticeably worse as indicated by the large portion of loop D’–E’ and parts of β-strands A’, C’ and F’ protruding out of the TV density (Figure 2C). Another difference was found in the P1-interface loop region (Figure 2B and 2C red arrows), where TV had more densities extending from the P1 subdomain to the most exposed exterior surface, implying that the P1-interface loop of TV is potentially longer than those of rNV and SMSV. An atomic or near-atomic resolution structure of TV will be required to better resolve the structural details of TV in the P1 and P2 subdomains.

### TV is Structurally Most Similar to rNV in the *Norovirus* Genus

**Figure 3:**
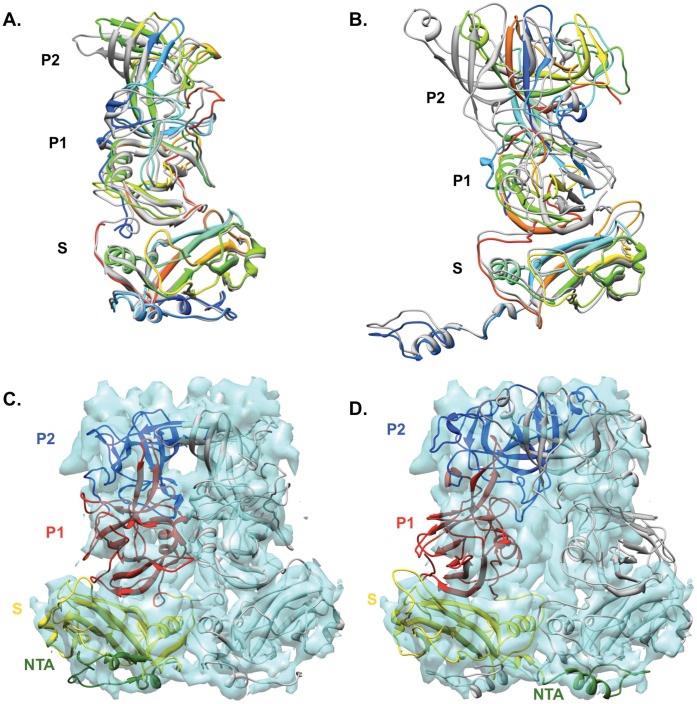
The S-P1-P2 inter-domain orientation and the dimer formation of TV. (**A**) The superimposition of original rNV (PDB ID: 1ihm chain b colored in gray) with its S domain (rainbow colored), P1 (rainbow colored) and P2 subdomains (rainbow colored) independently fitted into TV. Similar superimposition of SMSV is shown in (**B**). The TV A/B dimer density fitted with the A/B dimers of rNV and SMSV, are displayed in (**C**) and (**D**), respectively. The amino-terminal arm (NTA), S domain, P1 and P2 subdomain in B subunit of rNV and SMSV are colored in forest green, yellow, red and blue, respectively. The A subunits are colored in gray.

The flexible hinge (S-P1 hinge) linking the S domain and the P1 subdomain allows different relative orientations between the S and P domains among caliciviruses [3], [4], [6], [9], [12], [38]. There were only small changes of the rNV S, P1, P2 subdomains orientation and position after being individually fitted to TV densities relative to the result of whole subunit fitting (Figure 3A). However, the SMSV P1 and P2 subdomains underwent significant orientation changes when fitted individually (Figure 3B). The relatively minor changes in the orientation of rNV S, P1, P2 subdomains before and after being individually fitted into TV density and the significant alteration in that of SMSV indicate that TV has its P1 and P2 subdomains oriented with respect to the S domain more similarly to that of rNV.

The dimeric interface mediated by the P domains of the capsid dimers is suggested to be another variable among caliciviruses [6]. For rNV, both the P1 and P2 subdomains are involved in the dimeric interactions within the A/B and C/C dimers (Figure 3C), while in SMSV, the P2 subdomains of A/B and C/C dimers primarily mediate the dimeric interactions (Figure 3D). TV appeared to have both P1 and P2 subdomains involved in mediating the formation of the A/B dimers, essentially resembling the formation of rNV A/B and C/C dimers. Considering both the inter-domain orientations within the VP1 monomers and also the dimeric interface of the A/B capsid dimers, TV was found to be structurally most similar to Norwalk virus, the prototype of *Norovirus* genus, than to SMSV and other known calicivirus structures. This conclusion is also consistent with the phylogenetic analysis based on the amino acid sequences of calicivirus VP1 proteins [1].

### The P Domains of TV C/C Dimer are Highly Flexible

**Figure 4:**
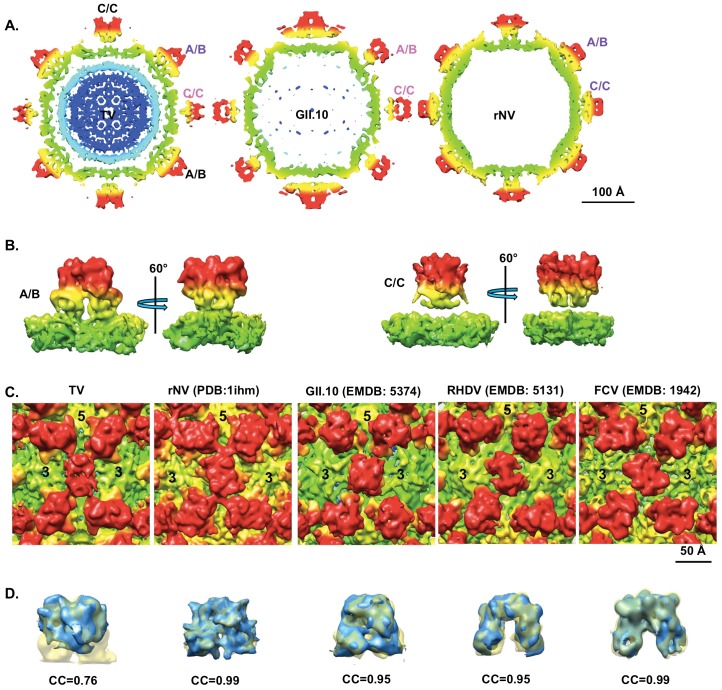
The flexible conformation of TV C/C dimers. (**A**) Central sections (5 Å-thick) of the 10 Å low-pass filtered TV map (left), a 10 Å GII.10 norovirus VLP (middle, EMDB: 5374), and a simulated map of rNV at 10 Å (right). (**B**) Densities for the A/B and C/C dimers were segmented from the 10 Å TV map, respectively, and are displayed with their S domains in the same views. (**C**) A zoom-in view near the icosahedral 2-fold axis of TV, rNV (simulated map), GII.10 NoV (EMDB: 5374), RHDV (EMDB: 5131), and FCV (EMDB: 1942). All the maps were filtered to the same level as RHDV by matching the 1-D structure factor. Maps in (A), (B) and (C) were radially colored using the same color key as in Figure 1. (**D**) The P dimers of A/B (yellow) and C/C (blue) from the equally filtered maps of TV, rNV, GII.10 NoV, RHDV and FCV were aligned together. The correlation coefficients (CC) between them were calculated in Chimera using “measure correlation” command [28].

Despite the well-resolved A/B dimers in the TV structure, TV had very flexible C/C dimers as evidenced by the diffuse weaker densities (Figure 1B, 1D). This is in stark contrast to the similarly well resolved A/B and C/C dimers in most other caliciviruses with known structures. Additionally, TV C/C dimers, compared to the A/B dimers, had significantly reduced S-P1 interactions as shown in Figure 4B. A clear gap between the S domain and the P domain was observed in the TV map that was low-pass filtered to 10 Å resolution to render weaker densities more clearly (Figure 4A left and 4B). The apparent gap between the S and P domain in TV CC dimers is reminiscent of the extended conformation of the A/B and C/C dimers of GII.10 NoV (Figure 4A middle) and MNVs (maps not shown) in which the P domain is elevated from S domain [9], [12]. Other than the difference in the S-P1 interactions, the overall shape of the P domains of C/C dimers also differed significantly from those of A/B dimers in TV and the A/B and C/C dimers of rNV (Figure 4A right).

As with many other T = 3 viruses [3], [4], [5], [39], [40], the S domains of A/B and C/C dimers of caliciviruses adopt “bent” ad “flat” conformations, respectively, to facilitate the formation of the closed icosahedral shell. The resulting structural difference between P domains of A/B and C/C dimers is usually subtle and nearly identical structures for the P domains of A/B and C/C dimers have been observed in almost all the calicivirus structures. However, in TV, noticeable difference was observed for the P domains of A/B and C/C dimers from the map surface rendered even at a lower resolution of ∼10.5 Å (Figure 4C). To quantify the similarity of A/B and C/C dimers in the P domains, we calculated the correlation coefficients between the P domains of C/C and A/B dimers of multiple calicivirus structures (Figure 4D). As expected, the correlation coefficients for most of the caliciviruses (rNV, FCV, GII.10 NoV and RHDV) were larger than 0.95 suggesting nearly identical structures for the P domains of A/B and C/C dimers in these viruses. However, the correlation coefficient for the P domains of TV A/B and C/C dimers was much lower (0.76), suggesting significant difference between the P domains of TV A/B and C/C dimers. From the apparently weaker densities for the P domains of C/C dimer, such differences most likely originated from a more flexible conformation of the P domains of TV C/C dimer, which resulted in smeared weaker densities in the map. The reduced inter-domain interactions between the S and P domains of C subunits could be a reason for the flexible P domains of TV C/C dimers.

### The NTAs of Subunits A, B and C and their Potential Interactions with the Inner Shell (IS)

**Figure 5:**
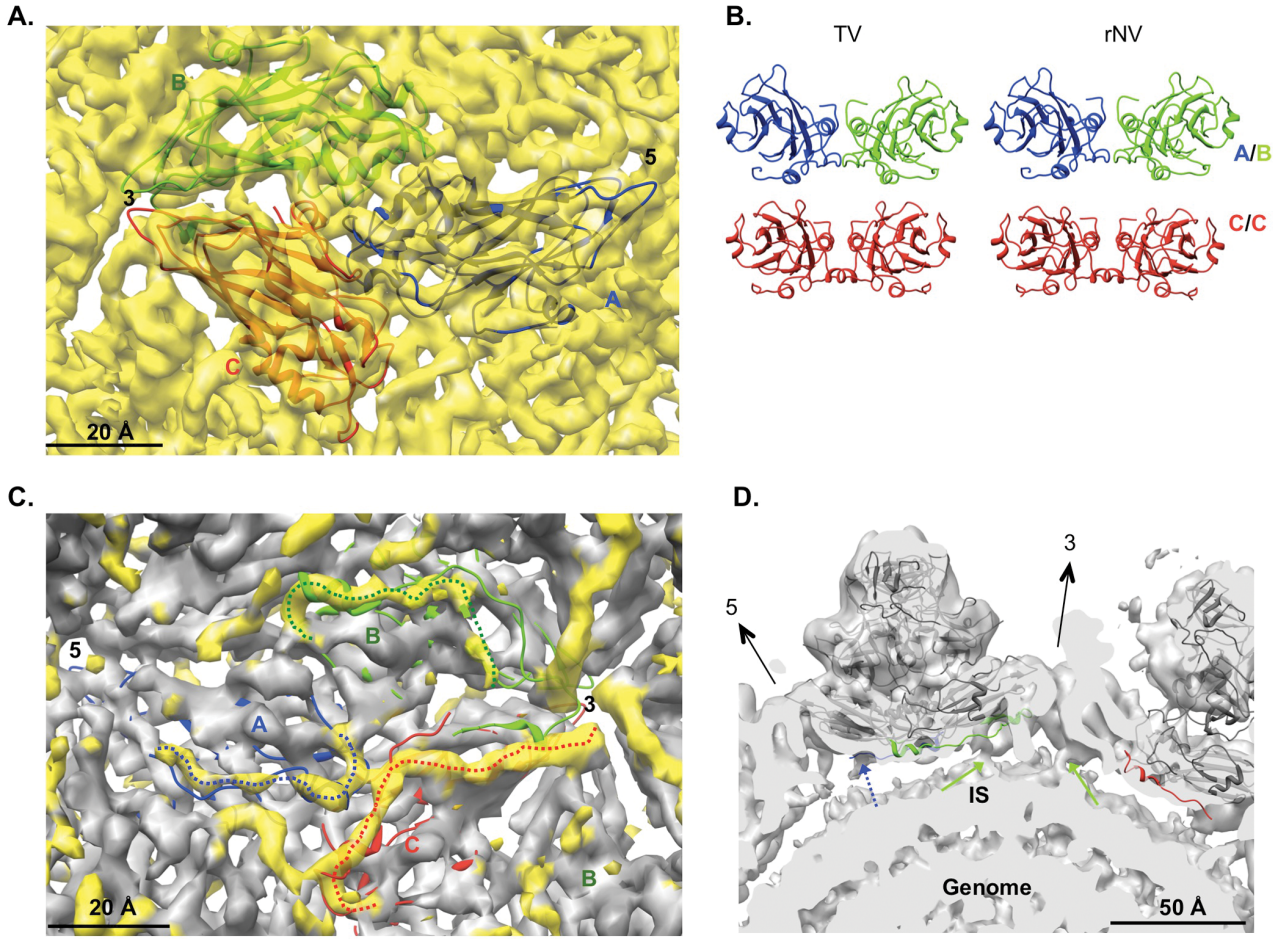
The icosahedral packing of S domains and the configurations of NTAs. (**A**) The top view of the icosahedral shell of TV and the fitting with rNV S domains of subunits A (blue), B (green) and C (red). Panels in (**B**) display the S domains within A/B and C/C dimers of rNV before (right) and after (left) independently fitted into TV density (see more details in Methods) (**C**) An inside view of TV icosahedral shell fitted with rNV S domains of subunits A (blue), B (green) and C (red). Regions fitted with rNV S domains (except the NTAs) are colored in gray, and the extra yellow colored densities corresponded to the NTAs of TV. Three dashed lines (blue, green and red) highlight densities for the NTAs of TV subunits A, B and C, respectively. (**D**) A section view of the 10 Å low-pass filtered TV map indicated connections between the S domain layer and the inner shell (IS) layer. The green arrows point to the relatively strong connections near the subunit B, and the dashed blue arrow indicates the weaker connection close to subunit A, which would be more obvious at a slight lower contour level.

The S domains of rNV A, B and C subunits were fitted into their counterparts in TV. In contrast to the P domains for which reliable fitting of calicivirus crystal structures into TV densities was only achievable for the well-resolved A/B dimers but not the flexible C/C dimers, the S domains of all the subunits A, B and C in TV were equally well-resolved and fitted similarly well with the atomic model of rNV (Figure 5A). Moreover, there were little changes in the relative positions of the S domains in subunits A, B and C of rNV before and after being fitted into TV density. These fitting results indicate that the S domains of the A, B and C subunits in TV are packed with respect to the icosahedral axes in a way very similar to that of rNV, with the A/B and C/C dimers also adopting the “bent” and “flat” conformations, respectively (Figure 5B). However, TV showed a distinct alteration in the amino-terminal region, which is often referred to as the N-terminal arm (NTA). NTAs, ∼30–50 residues at the N-terminus of the capsid proteins, are suggested to be important molecular switches that determine the conformation of A/B and C/C dimers and control the assembly of caliciviruses [3], [4], [7] and many other T = 3 viruses [39], [40], [41]. Significant variations, including the number of ordered and visible residues in different subunits, and the arrangement of the NTAs beneath the icosahedral shell, have been observed among NTAs of caliciviruses from different genera [3], [4], [5], [10]. In the rNV structure, the NTA of B subunit is almost fully resolved while more than half of the NTAs of A and C subunits are disordered. From the fitted rNV structure in the TV density (Figure 5C), both TV and rNV had the NTAs of the A subunits converge at the icosahedral 5-fold axes whereas NTAs of the B and C subunits extended towards the icosahedral 3-fold axes. The NTA of the TV subunit A was nearly superimposed with the NTA of rNV A subunit, probably with a similar number of ordered residues and an almost identical extending direction. However, the NTAs of the TV B and C subunits differed from the corresponding NTAs of rNV in both the number of resolved residues and the extending directions at the distal ends. In the TV structure, the NTA of C subunit is the best resolved in contrast with rNV, in which the B subunit is resolved best. Although the NTAs of the B and C subunits in TV and rNV exhibited opposite configurations, they both appeared to employ interactions between the neighboring B and C subunits to acquire and stabilize the “flat” conformation of the C/C dimers. For rNV, the long ordered NTAs of the B subunits extend to the neighboring C subunits to mediate the formation and stabilization of the “flat” conformation of C/C dimers [3]. Likewise, TV had the longer C subunit NTAs interacting with the neighboring B subunit to switch the capsid dimers to the “flat” conformer (Figure 5C). In contrast to both TV and rNV, the NTAs of SMSV were nearly fully resolved for all the subunits, with NTAs of the A and C subunits converging at the icosahedral 3-fold axes while NTAs of B subunits centered around the icosahedral 5-fold axes [4].

In the 10 Å-low pass filtered map of TV, connections between the S domain shell and the inner shell beneath could be observed near the icosahedral 3-fold axes and also at the 5-fold axes (at a slightly lower contour level) (Figure 5D). Similar connections have been previously reported in a cryo-EM structure of the SMSV virion [6] and could also be found in the FCV virion (EMDB: 1942). Thus, it is likely that these connections should also be present in other calicivirus virions. Using the fitted rNV crystal structures as reference, stronger connections were seen below the S domains of TV B subunits, and weaker connections could be observed beneath the A subunits, while connections between the C subunits and IS were much less evident (Figure 5D). Since the stronger connections were found close to the NTAs of the A and B subunits of TV, it is possible that a portion of the disordered N-terminal residues in the A and B subunits are involved in the interactions with the IS.

## Discussion

### TV is Potentially a More Appropriate Surrogate for Human NoVs

Human NoVs, the major cause of acute gastroenteritis pandemics, are currently understudied due to the lack of efficient *in vitro* culture systems and animal models. Our structure revealed that TV is a calicivirus with both conserved and distinct features. In terms of the overall architecture, TV exhibited the characteristic “arch-like” surface density and 32 large hollows at icosahedral 3- and 5-fold axes; the capsid protein of TV had the conserved fold of caliciviruses in both the S and P domains, all of which supported the classification of TV into the *Caliciviridae* family. In addition, previous structure comparison among the caliciviruses has suggested that viruses from different genera show more pronounced changes in the S-P1-P2 inter-domain orientations than viruses within the same genus [6]. Thus, the fact that the TV A and B subunits exhibited similar S-P1-P2 orientation with rNV further confirmed the close relationship between TV and NoVs, previously indicated by the amino acid sequence comparison [1]. In addition to these significant structural similarities, TV utilizes HBGAs as cellular receptors, as do human NoVs [15]. Furthermore, TV infects primate rhesus monkeys which, compared to mice, are evolutionarily more closely related to humans. All these properties make the cultivatable TV an attractive surrogate for human NoVs that are still recalcitrant to *in vitro* cultivation.

### The Origin and the Role of the Highly Flexible P Domains of C/C Dimers in TV

Another interesting observation of the TV structure was that the P domains of C/C dimers were highly flexible and perhaps more flexible than most, if not all, other caliciviruses with known structures based on the cross correlation analysis on P domains of A/B and C/C dimers (Figure 4D). This raises the questions of how TV C/C dimers obtain this highly flexible conformation for the P domains and why TV needs such flexibility for its C/C dimers. The significantly reduced S-P1 interaction within the C subunits is possibly the direct cause for the flexible P domains of C/C dimers, but how this S-P1 inter-domain interaction reduction occurs is ambiguous. It is possible that the P domains of TV C/C dimers are elevated from the S domains, probably similar to MNV and GII.10 NoV [9], [11], [12]. However, unlike MNV and GII.10 NoV, in which the P domains of both A/B and C/C dimers are lifted and form a new network of inter-dimer interactions between the P domains of neighboring dimers to stabilize the structure [9], [11], [12], the elevated P domains of TV C/C dimers alone lack such inter-dimer stabilizing contacts and become highly flexible.

A recent cryo-EM study on the cultivatable FCV complex with fJAM-A, its functional cell receptor, reveals that such virus-receptor interactions can initiate distinct conformational changes in the A/B and C/C dimers of FCV, and the poorly resolved C/C dimers are possibly in a more flexible conformation than A/B dimers in the virus-receptor complex [38]. Thus, the significantly flexible P domains of TV C/C dimers imply that the TV virion particles are likely to be in a pre-primed state for interaction with the host cell. Moreover, since the C/C dimers displayed the most significant deviation between TV and the non-cultivatable Norwalk virus structure (rNV, PDB ID: 1ihm), it is tempting to propose that the flexible conformation of TV C/C dimers is a critical factor for the propagation of TV in the cell culture. It is likely that conformational changes of the capsid protein dimers, in particular the enhanced flexibility of the P domains of C/C dimers, are necessary for the exposure of some buried residues important for the efficient virus-cell interactions and the following steps of uncoating and genome release of caliciviruses. This hypothesis is supported by the suggested involvement of buried residues in FCV-receptor interaction [5] and GII NoVs interaction with antibody [12]. Though the complete cell entry of TV might require additional factors (e.g., cell receptors), the pre-primed flexible state of TV C/C dimers could facilitate interactions with host cells and contribute to the successful *in vitro* propagation of TV.

### The S-P1 Hinges and Resistance of Caliciviruses to *in vitro* Propagation

The conformation changes of caliciviruses are directly related to the flexibility of the S-P1 hinge between the S domain and the P1 subdomain as revealed by the “extended” conformation of MNV and GII.10 NoV [9], [12], and the twist and tilt of P domains in FCV upon receptor binding [38]. The flexible S-P1 hinge may be also responsible for the significant flexibility of P domains of TV C/C dimers. A previous study of FCV has suggested the importance of the S-P1 hinge, especially the residue G329, in interactions with soluble fJAM-A receptor and the receptor-induced conformational changes [5]. Multiple sequence alignment of the S-P1 hinge region of TV, NoVs, Vesiviruses and Lagovirus revealed a clear pattern of residues of different chemical properties at the reported critical residue G329 of FCV and the corresponding position of other caliciviruses (Figure S2). Either Gly or non-polar Ile is at this position for the cultivatable caliciviruses, while polar Thr or Ser is at the same position for non-cultivatable caliciviruses. This clear residue pattern further suggests the importance of residue at this position. Nevertheless, more studies are needed to further elucidate whether the residues in the S-P1 hinge are truly related to human NoVs’ resistance to *in vitro* cultivation.

### Conclusion

In conclusion, we have determined a 6.3 Å cryo-EM structure of TV, which revealed its close relationship with NoVs. Comparative analysis between TV, NoVs, Vesiviruses and Lagovirus indicated the importance of conformational flexibility of caliciviruses (i.e., the highly flexible P domains of C/C dimers) to the virus-host cell interactions and the consequent cell entry of caliciviruses. Further investigations on viral assembly and virus-cell interactions are required to better understand the obstacles in the propagation of human caliciviruses, especially human NoVs.

## Supporting Information

Figure S1 **Characterization of TV purified from CsCl gradient.** The peak fractions (fractions 6–8) of the CsCl gradient containing TV were identified by the detection of the major structural protein VP1 (∼57 kDa) by SDS-PAGE (A) and Western blot analysis (B). Infectious virus was detected by the plaque assay (C). Purified TV from fraction 7 also demonstrated hemagglutination with type B human red blood cells (D).
(TIF)

Figure S2 **Multiple sequence alignment on the S-P1 flexible hinge region.** Sequences of TV, NoVs, Vesiviruses and Lagovirus were obtained from NCBI GenBank, and aligned using the ClustalW2 online server. The figure was prepared in Chimera program and the amino acids sequences were color using the “Clustal X” scheme. The arrow indicates residue G329 in FCV and the corresponding residues in other calicivirus strains. The asterisks (*) point to the conserved “FXXLXPP” motif in the S-P1 hinge. The residue ranges of TV (193–220) and NV (211–230) are labeled. Sequences above the long red line are from the non-cultivatable caliciviruses, and those below the red line are sequences of caliciviruses having permissive cell lines.
(TIF)
