# Supplementary figures and images for: Cryo-EM Structure of a Novel Calicivirus, Tulane Virus
Source: PLoS One. 2013 Mar 22;8(3):e59817. doi: 10.1371/journal.pone.0059817 (PMC3606144; doi:10.1371/journal.pone.0059817)

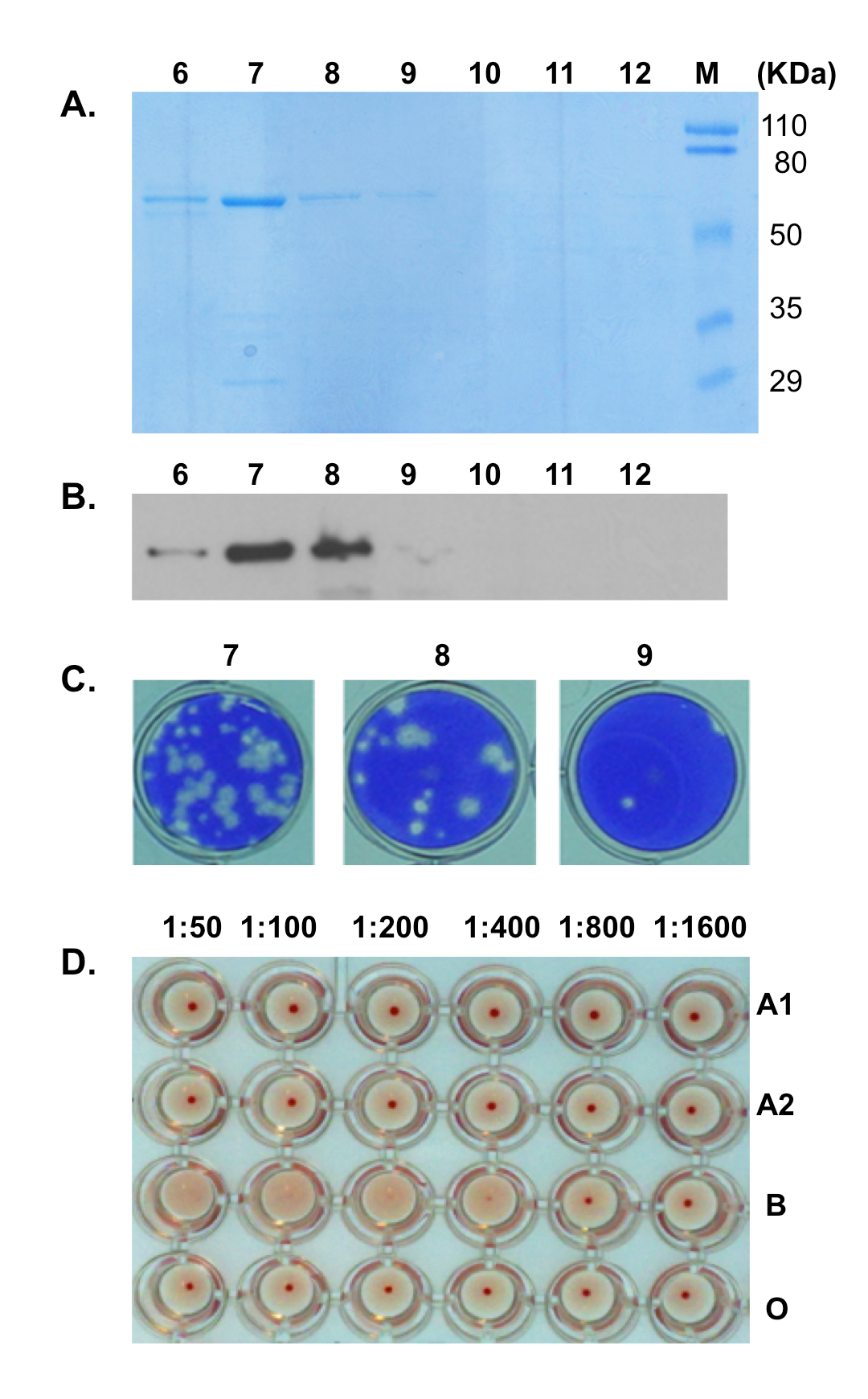

Supplement: Figure S1 — Characterization of TV purified from CsCl gradient. The peak fractions (fractions 6–8) of the CsCl gradient containing TV were identified by the detection of the major structural protein VP1 (∼57 kDa) by SDS-PAGE (A) and Western blot analysis (B). Infectious virus was detected by the plaque assay (C). Purified TV from fraction 7 also demonstrated hemagglutination with type B human red blood cells (D). (TIF) [file pone.0059817.s001.tif]

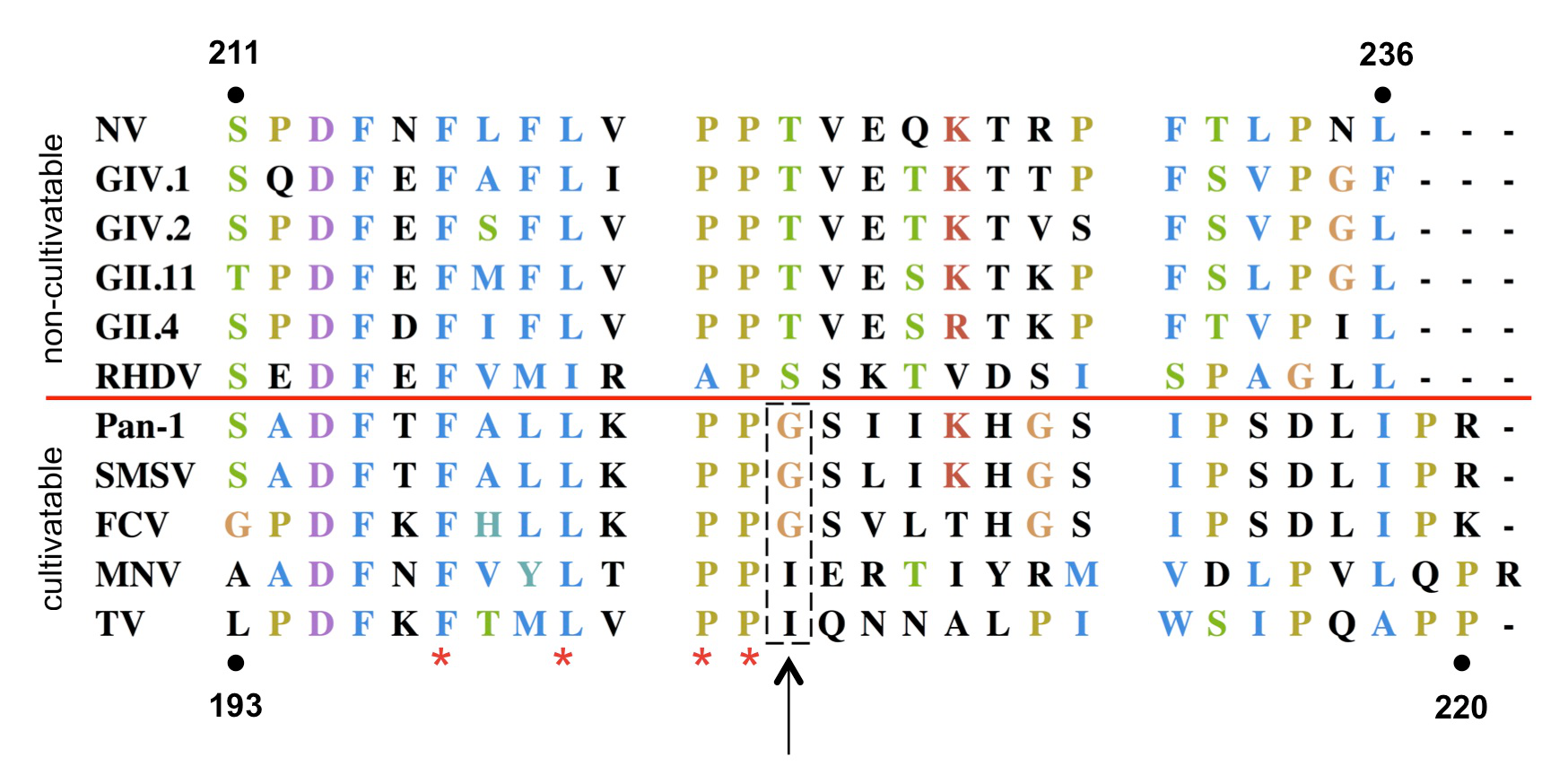

Supplement: Figure S2 — Multiple sequence alignment on the S-P1 flexible hinge region. Sequences of TV, NoVs, Vesiviruses and Lagovirus were obtained from NCBI GenBank, and aligned using the ClustalW2 online server. The figure was prepared in Chimera program and the amino acids sequences were color using the “Clustal X” scheme. The arrow indicates residue G329 in FCV and the corresponding residues in other calicivirus strains. The asterisks (*) point to the conserved “FXXLXPP” motif in the S-P1 hinge. The residue ranges of TV (193–220) and NV (211–230) are labeled. Sequences above the long red line are from the non-cultivatable caliciviruses, and those below the red line are sequences of caliciviruses having permissive cell lines. (TIF) [file pone.0059817.s002.tif]
